# Miscellaneous

**Published:** 1893-03

**Authors:** 


### MISCELLANEOUS.

### PRINCE GEORGE AND THE BLUEJACKET.

When Prince George, Duke of York, had the independent command of the Thrush, on the West Indian station, it fell to his lot to have to convey, as prisoner, a young bluejacket belonging to another ship, who had been hitherto a constant offender and continually on the blacklist. The man came on board the Thrush merely as a prisoner for conveyance from one part of the station to another under sentence of punishment. From his demeanor, however, and by close observation of him, Prince George came to the conclusion that there were many seeds of good in the man and the making of a better career. When the term of punishment was fulfilled, and the time came for him to rejoin his own ship, Prince George determined to try and give him the chance of a new start in life. On arriving in port, after calling upon the man's captain, who naturally was only too glad to be rid of him, he went to the Admiral and asked permission to transfer him to the Thrush. The Admiral, astonished at the proposition, gave his consent. Prince George went back to his own ship, had the man brought aft before him on the quarterdeck and spoke to him as probably he had never been spoken to before. He told him that he was henceforth transferred to the Thrush; that, as commanding officer, he put him in the first class for leave and gave him a clean sheet as regards his past offences. "I do not ask you to make me any promise as to your future behavior. I trust to your honor and good feeling alone. But remember that, by the rules of the service, if you offend again in any way, or break your leave, I have no option, but am bound to put you straight again to that class from which I now remove you. Your future is in your own hands. You have had no leave for twelve months. Go ashore now with the special leave men. Your pay has been stopped, and no money is due to you. Here is a sovereign. I trust to you not to misspend it. You know as well as I do what you may do, and what you may not do. God help you to do the right and keep you from wrong." The man was completely overcome. He, of course, answered nothing, but saluted and was then marched forward again. His commanding officer's confidence was not misplaced. During the rest of the Thrush's commission he was never once an offender, but showed himself as active, willing and smart a hand as any in the ship, and after working hours he was the life of the forecastle. In the ship in which he has subsequently served he has maintained his good conduct and attained a petty officer's rating.

---

### ABOUT SLANG.

" Slang is no good," remarked the drummer, "and a man is clean off his base that monkeys with it."

" As to how ?" inquired the hotel clerk.

" Well, this is how," continued the drummer. "Two weeks ago I went into Boston with a traveller from Chicago who couldn't speak ten words of English pure and undefiled. At the railroad station he tackled a hack driver.

" 'Say, hackie, what's the damage to take me to Parker's ?'

" 'No damage at all, sir,' replied the hackman, with a Harvard accent.

" 'Sure ?'

" 'Quite sure, sir, if you go with me.'

" 'Then I'll go with you,' and he piled in, and I took a street car.

"At Parker's the driver opened the door politely and landed him out.

" 'Are you all right, sir ?' he inquired.

" 'You bet I am. Never was finer '

" 'Had a pleasant drive ?'

" 'Bang up.'

" 'Arrived here in good condition ?'

" 'Al, thanks,' and he started into the hotel.

" 'Two dollars, please,' said the driver, detaining him.

" 'I thought you said there was no damage ?' he said in surprise.

" 'There wasn't any, sir. Haven't you just said everything was all right, and you were in excellent condition.'

" 'Yes, but—'

" 'I understand perfectly, sir," interrupted the driver ; 'if you had asked the fare I should have informed you, but you did not. I presumed that it was quite immaterial to you so long as you were delivered at your destination safely and without damage.'

" The slangy man did'nt discuss the point. He handed over the two dollars, and has since been more careful of his slang."

---

A Wonderful Set of Chessmen.—A remarkable set of chessmen has just been completed by an American mechanic. The pieces are made of silvered bronze, and the period of costumes and equipments is A. D. 1194, all the characters being historical and contemporary and strictly accurate in every detail of heraldic blazonry and costume. The knights are in chain-mill armor, with shield, ax, sword, and dagger. Their fur coats have each the individual blazon of the wearer. The queens wear royal robes and carry scepters. The bishops are in church vestments, with cross and crozier. The pawns are men at arms in a kneeling posture, with spear, bill-hook and knife. The white men are English, the black French. The English king and queen are Richard I. and his berengaria. The bishops are Herbert Walter, Archbishop of Canterbury, and William Longchamps, Bishop of Ely ; and the knights are the Earl of Salisbury and Baron of Worcester. The castle is Anglo-Norman, and is a perfectly accurate representation of feudal architecture. The French king and queen are Philip and Ingeborg, his Danish spouse, the bishops being De Dreux and De Sully, of Beauvais and Paris. The knights are also well-known men of the twelfth century, and the castle is Franco-Norman. The set has taken upwards of six years to make.

A Man Eater with 149 Known Victims.—The panther has always been distinguished for its cunning and bloodthirsty ferocity, and unrivalled in these respects is the record of "Aroni Lalpur man eater," killed this year in India. This animal used generally to lie in wait for his victims near villages, concealed in a tree, and was eventually killed after considerable difficulty by a party of sportsmen beating with a line of eighteen elephants. It took, however, several shots to kill him, and he received eight wounds before succumbing. Some idea may be formed of the taste for human blood exhibited by this feline scourge. In 1890, he killed eight people ; during 1891 and 1892 he was credited with the following : 1891—January, 8 ; February, 2 ; March, 6 ; April, 1 ; May, 7 ; June, 6 ; July, 7 ; August, 14 ; September, 12 ; October, 12 ; November, 10 ;

December, 13. 1892—January, 14; February, 21; March, 8. Of these sixty were children under ten years of age, and forty were women.

THE FIRST UNITED STATES POST-OFFICE.—In the English colonies, which afterward became the United States, a postal system was projected as early as 1692, but owing to the thinness of the population it was not organized until 1710. By act of parliament chief offices were established in New York and other important places in the colonies. In 1753, Benjamin Franklin was appointed deputy postmaster general for the colonies and under him the postal system was greatly improved. The first mail coach ran once a week between Philadelphia, a stage wagon starting from each city Monday morning and reaching its destination by Saturday night. In 1789 the constitution of the United States conferred upon congress the exclusive control of postal matters for all the states. The post-office department was one of the first organized, says the Chicago Inter Ocean, from which we quote.

